# Factors associated with smartphone addiction: A comparative study between Japanese and Thai high school students

**DOI:** 10.1371/journal.pone.0238459

**Published:** 2020-09-08

**Authors:** Arunrat Tangmunkongvorakul, Patou Masika Musumari, Yukie Tsubohara, Pisittawoot Ayood, Kriengkrai Srithanaviboonchai, Teeranee Techasrivichien, S. Pilar Suguimoto, Masako Ono-Kihara, Masahiro Kihara

**Affiliations:** 1 Research Institute for Health Sciences, Chiang Mai University, Chiang Mai, Thailand; 2 Global Health Interdisciplinary Unit, Center for the Promotion of Interdisciplinary Education and Research, Kyoto University, Kyoto, Japan; 3 Sankampang Hospital, Chiang Mai, Thailand; 4 Department of Community Medicine, Faculty of Medicine, Chiang Mai University, Chiang Mai, Thailand; 5 Department of Health Informatics, Kyoto University School of Public Health, Kyoto, Japan; 6 Center for Medical Education, Graduate School of Medicine, Kyoto University, Kyoto, Japan; National University of Singapore, SINGAPORE

## Abstract

**Background:**

Smartphone addiction is a growing social problem with adverse health outcomes. There are few comparative studies in Asia that examine factors associated with smartphone addiction. The current study aimed to address this research gap by presenting a comparative analysis of factors associated with smartphone addiction in Japan and Thailand, two countries heterogeneous in both their level of economic development and culture.

**Methods:**

Participant data were collected using two population-based surveys. Participants were high school students in grade 11, aged 16–17 years old, and were selected using quota sampling in Japan in 2014 and random sampling in Thailand in 2016. The outcome of interest was smartphone addiction, measured using a modified version of the Young Diagnostic Questionnaire for Internet Addiction. Multiple logistic regression analysis was performed to determine factors associated with smartphone addiction (gender; nationality; family connectedness; and average time spent on smartphone per day).

**Results:**

This study included a total of 7694 students, 6585 students from Japan and 1109 students from Thailand. The prevalence of smartphone addiction was 35.9% among Thai students and 12% among Japanese students. Thai students were more likely to have smartphone addiction than Japanese students (AOR 2.76; 95% CI: 2.37–3.30). Being female was associated with increased odds of smartphone addiction in both Japanese (AOR 1.53; 95% CI: 1.32–1.78) and Thai students (AOR 1.34; 95% CI: 1.01–1.78). The parental connectedness variables “my parents noticed when I was unhappy” (AOR 0.77; 95% CI: 0.62–0.96) and “my parents noticed when I did something good” (AOR 0.78, 95% CI: 0.61–0.99) were associated with lower odds of smartphone addiction among Japanese students.

**Conclusion:**

Smartphone addiction was more prevalent among Thai adolescents than Japanese adolescents, and more prevalent among females than males in both countries. Interventions for reducing smartphone addiction should take into account both context and gender, and should leverage the protective effect of parental connectedness.

## Introduction

In just a few decades, smartphones have become ubiquitous devices in both developed and developing settings, and across populations of different age strata. The rapid adoption of these devices have been attributed to their portability and ability to provide a variety of functions such as Internet browsing, access to email, easy access to social media networks, real-time broadcast, camera, navigation system, and multimedia players. The global number of smartphone users has increased from 1.57 billion in 2014 to a current estimate of 2.71 billion [1].

Although smartphones have transformed lives in positive ways, such as increased productivity and social networking, there is growing evidence indicating that people overuse their phones in ways that interfere with their daily lives and mental health [2, 3]. Various terms have been used to describe different patterns of smartphone overuse, such as “smartphone addiction”, “problematic smartphone use”, and “excessive smartphone use” [4]. This study will refer to this phenomenon as smartphone addiction. Smartphone addiction has rapidly grown to become a major social and health problem, particularly among adolescents and young people. A recent systematic review examined the prevalence of problematic smartphone use, including smartphone addiction, and found a prevalence between 10% and 30%, with a median of 23.3% [5]. In Asia, the geographic region of this study, the prevalence of smartphone addiction and other types of smartphone overuse among adolescents and young people varied across studies and countries. For example, studies have documented prevalence ranging from 13.5% to 36% in South Korea [6–12], 4.05% to 29.8% in China [13–23],15.5% to 82% in India [24–27], and 62.6% in Filipino adolescents [28]. In Japan and Thailand, the focus of the current research, very few studies have reported on the prevalence of smartphone addiction. One Thai study found that 45.8% of students aged 18–24 years in Chiang Mai had excessive smartphone use [29], while in Japan, Tateno and colleagues documented a smartphone addiction prevalence of 22.8% among males and 28% among female college students (mean age 19 years ±1.3) [30]. In Western countries, the prevalence of smartphone addiction and/or other problematic smartphone use ranged from 16.9% to 43.3% [31, 32].

Many factors are associated with smartphone addiction and other patterns of smartphone overuse among adolescents and young people in Asia. These include for example, depression and anxiety [7, 8, 14, 19, 20, 33], loneliness and shyness [13], low psychological well-being [29], low self-esteem [34], substance use (alcohol use and smoking) [19, 33, 35], poor sleep quality [12, 14, 20], low academic performance [9, 27, 35], being female [9, 11, 33, 35–38], having a poor relationship with parents [9], less involvement at school and less satisfaction with school life [9], and negative parenting style [39].

A recent cross-cultural study comparing smartphone use between Chinese and British students found that Chinese students were more likely than British students to have problematic smartphone use [32]. Asian countries are heterogeneous in their level of economic development and culture, which can potentially influence patterns of smartphone use. However, there are few bi-national or multi-country studies comparing the prevalence of and factors associated with smartphone addiction among adolescents in Asia despite the growing research related to smartphone use in the region. To address this research gap, the present study explored the prevalence of smartphone addiction and factors associated with smartphone addiction among Japanese and Thai in-school adolescents.

## Materials and methods

### Participants

The present study included participant data from two of our population-based surveys conducted in Japan and Thailand. The sample from Japan was obtained from the 2014 Well-being of Youth in Social Happiness (WYSH) survey. The survey was part of the WYSH Project which was designed to prevent HIV and other sexually transmitted infections, and to promote the well-being of youth in Japan. The 2014 WYSH survey targeted male and female second year public high school students (grade 11), aged 16–17 years old. The participants were recruited using quota sampling. This sampling method provided the most cost-effective means to obtain a representative sample of second year students in Japan based on feasibility and availability of resources. Quotas were established based on the nine geographic regions of Japan (Hokkaido; Tohoku; Kanto; Tokyo; Hokushinetsu; Tokai; Kinki; Kyushu; Chushikoku) and types of schools (standard or specialized/comprehensive). We selected five schools in each of region (consisting of three standard schools and two specialized or comprehensive high schools), resulting in a total of 45 schools from all over Japan. In calculating sample size, Internet addiction was used as a proxy for smartphone addiction because very little data on smartphone addiction existed at the time the study was conducted. Assuming an Internet addiction prevalence of 6% among boys and 10% among girls in middle schools in Japan [40], a total sample size of 1542 (771 per group) was necessary to detect a 4% difference in the prevalence of Internet addiction between genders with a statistical significance at α = 0.005 and power of 80%. To ensure there was enough precision for multiple logistic regression analyses (15 outcomes for a maximum of 15 variables in the model; the rule of thumb being at least 10 outcomes were needed for every independent variable in the multiple logistic regression model [41]), subgroup analyses by gender or region (doubling the sample size), and possible non-response (15% of the sample), we decided to recruit 7200 students selected from four second year classes, each with at least 40 students from each school (160 students per school for the 45 schools included in this study).

The sample from Thailand was obtained from the Thai version of the WYSH study, which was conducted in 2016. The Thai survey included similar items to those employed in the 2014 WYSH survey in Japan. The survey similarly targeted male and female second year high school students (grade 11), aged 16–17 years old. Unlike the Japanese study, the sample for the Thai study consisted of students from only one geographic location, namely Chiang Mai, Northern Thailand. Researchers in Thailand did not have the resources to conduct a national study and therefore focused recruitment in an urban area of Chiang Mai province. The participants were recruited from all the fifteen schools located in Chiang Mai City. All participants from a set of randomly selected classrooms in each of the schools were invited to participate in the study. We estimated a required sample size of approximately 400 male and female students each based on Krejcie and Morgan’ formula [42]. The number is based on an estimated population size of 1696 male and 2283 female grade 11 students in Chiang Mai and factoring in a 5% margin of error, the proportion of the population with a given attribute was set at 0.5 (because this would provide the maximum sample size). The chi-square value for one degree of freedom was set at a 95% confidence interval (3.841).

### Data collection and variables

Data were collected using a paper-based self-administered structured questionnaire. The following were variables in the present study: smartphone addiction, other smartphone and social media-related variables, family connectedness, and socio-demographic variables. The Japanese and Thai questionnaires were each pilot tested among 15 and 20 students to assess readability and understanding. The wording of the questionnaire was modified accordingly. The survey in Japan was conducted either in students’ homerooms or other locations as designated by school authorities in Japan and in students’ classrooms in Thailand.

#### Smartphone addiction

We adapted the Young Diagnostic Questionnaire for Internet Addiction by replacing the variable “Internet use” with “smartphone use,” since the original scale used “Internet use” to denote all types of online activity [43]. The scale consisted of eight items, scored as 0 (“no”) and 1 (“yes”). Sample items included: i) you feel preoccupied with your smartphone; ii) you feel the need to use your smartphone for an increasing amount of time in order to achieve satisfaction; iii) you have repeatedly made unsuccessful efforts to control, cut back, or stop smartphone use; and iv) you spend more time on your smartphone than originally intended. The total possible score ranged from 0 to 8. Participants who answered "yes" to five or more of the criteria were classified as addicted smartphone users and the remainder were classified as non-addicted smartphone users [43]. The Young Diagnostic Questionnaire for Internet Addiction has been validated in both Japan and Thailand [44, 45]. The overall Cronbach’s alpha of the original Young Diagnostic Questionnaire for Internet Addiction based on a systematic review of 11 studies comprising a total of 6821 participants was 0.889 [46]. The modified questionnaires in our study had a Cronbach’s alpha of 0.76 for the Japanese sample, 0.70 for the Thai sample, and 0.77 for the pooled sample.

#### Other smartphone and social media-related variables

These variables included: i) ownership of a smartphone; ii) time spent on smartphone per day; iii) use of social networking sites; and iv) restriction of smartphone use by parents.

#### Family connectedness

A range of variables related to family connectedness was collected. These included: (i) frequency of dining with family in the past week; (ii) frequency of talking with parents; iii) satisfaction with relationship with father; iv) satisfaction with relationship with mother; v) perceived love and care from parents; and vi) two parental involvement variables (“How often do your parents notice when you have a problem/are not happy?”; “How often do your parents notice when you do something good?”)

#### Socio-demographic variables

The main socio-demographic variables included gender (female and male) and nationality (Japanese and Thai).

### Ethics statement

In Japan, the study was approved by the National High School PTA (Parent-Teacher Association), while the Human Experimentation Committee at the Research Institute for Health Sciences, Chiang Mai University (Certificate of Ethical Clearance No. 48/2016) provided ethical clearance in Thailand. All participants received information about the study objectives, roles of participants, their right to choose to answer or not answer any question, confidentiality, and how study findings would be presented. Participants and legal guardians provided written informed consent prior to participating in the study.

### Statistical analyses

The analysis was performed using SPSS 17 (PASW) for Windows (SPSS Inc., Chicago, Illinois, USA). Bivariate analysis was conducted using Chi-square tests and unadjusted logistic regression to explore factors associated with smartphone addiction (gender; nationality; family connectedness; and average time spent on smartphone per day). Variables that were statistically significant at p<0.05 in the bivariate analysis were included in the multiple logistic regression analysis. The analysis was conducted separately for the Japanese, Thai, and then pooled samples. There was no evidence of multicollinearity.

## Results

### Characteristics of participants and smartphone use

This study included a total of 7694 students, 6585 from Japan and 1109 from Thailand. There were slightly more females than males (55.9% vs 44.1%) in the Thai sample, while the proportion of Japanese male and female students were almost equal. The proportion of smartphone ownership was very high in both populations, 95% and 97.5% in Japanese and Thai students respectively. The same trend was noted for social media use whereby 97.7% of Japanese students and 99.6% of Thai students reported having previously used social media. Both the proportion of smartphone ownership and social media use was significantly higher in Thai students than in Japanese students. Nearly half of Thai students (45.3%) used smartphones more than five hours per day with far fewer Japanese students doing so (12.8%). With regards to parental control over students’ smartphone usage, Japanese parents mostly relied on filtering programs (Japanese: 33.6%, Thai: 0.8%) and limitations imposed by monthly packages (Japanese: 18.1%, Thai: 9.6%), while Thai parents tended to impose restrictions on usage time (Japanese: 5.1%, Thai: 13.9%). In total, 35.9% of Thai students and 12% of Japanese students were categorized as having a smartphone addiction (Table 1).

**Table 1:** Smartphone use in Japanese and Thai high school students.

	Japanese (6585)	Thai (1109)	*p*-value
	n (%)	n (%)	
**Gender**			< 0.001
Female	3327 (50.5)	620 (55.9)	
Male	3258 (49.5)	489 (44.1)	
**Own a smartphone**	6245 (95.0)	1081 (97.5)	< 0.001
**Have used social media**	6379 (97.7)	1105 (99.6)	< 0.001
**Smartphone usage per day**			< 0.001
One hour or less	2068 (31.5)	136 (12.4)	
2–4 hours	3657 (55.7)	466 (42.4)	
5 hours and more	842 (12.8)	498 (45.3)	
**Parental measures to restrict smartphone use**			
Limit time usage	331 (5.1)	154 (13.9)	< 0.001
Limit through monthly package	1178 (18.1)	106 (9.6)	< 0.001
Set up filtering program	2189 (33.6)	9 (0.8)	< 0.001
**Smartphone addiction measures**			
I have been preoccupied with using my smartphone.	2992 (45.8)	674 (61.9)	< 0.001
I’ve extend usage time for my satisfaction.	2093 (32.1)	626 (57.4)	< 0.001
I’ve tried to reduce or quit using my smartphone but failed.	1616 (24.8)	528 (48.5)	< 0.001
I felt unhappy when I tried to reduce or quit using my smartphone.	806 (12.4)	316 (29.0)	< 0.001
I’ve spent more time using my smartphone than I intended.	2465 (37.9)	897 (82.1)	< 0.001
I’ve had relationship problems due to smartphone use.	420 (6.1)	332 (30.3)	< 0.001
I’ve lied to people to hide my smartphone use.	620 (9.5)	180 (16.5)	< 0.001
I’ve used my smartphone to escape from problems or feelings	1252 (19.2)	507 (46.9)	< 0.001
Smartphone addiction*	776 (12.0)	382 (35.9)	<0.001

^a^ *p-*values were calculated by χ2 tests.

*: “yes” to five or more of the above 8 items of smartphone addiction

### Family connectedness

More Thai students than Japanese reported engaging in activities indicative of family connectedness such as dining with family every day, always talking to parents, being very satisfied with their relationship with both parents, and feeling love and care from parents. Alternately, Japanese students were more likely than Thai students to have parents that noticed when they were unhappy or did something good (Table 2).

**Table 2:** Family connectedness of Japanese and Thai high school students.

	Japanese (6585)	Thai (1109)	*p*-value
	n (%)	n (%)	
**Frequency of dining with family in the past week**			< 0.001
7 days	2302 (35.4)	455 (45.1)	
5–6 days	647 (10.0)	107 (10.6)	
3–4 days	1025 (15.8)	188 (18.6)	
1–2 days	1509 (23.2)	178 (17.6)	
Never	1019 (15.7)	81 (8.0)	
**I talk to my parents**			< 0.001
Always	2828 (43.2)	614 (56.1)	
Often	1713 (26.2)	274 (25.0)	
Sometimes	1472 (22.5)	104 (9.5)	
Rarely	454 (6.9)	96 (8.8)	
Never	82 (1.3)	7 (0.6)	
**My parents notice when I have a problem or feel unhappy**			< 0.001
Regularly	3632 (66.8)	627 (59.9)	
Rarely	1367 (25.2)	312 (29.8)	
Never	435 (8.0)	107 (10.2)	
**My parents notice when I do something good**			< 0.001
Regularly	4705 (80.6)	769 (73.0)	
Rarely	945 (16.2)	225 (21.3)	
Never	187 (3.2)	60 (5.7)	
**Satisfaction with relationship with mother**			< 0.001
Very satisfied	2243 (35.0)	655 (60.1)	
Satisfied	2797 (43.6)	313 (28.7)	
Neither satisfied nor unsatisfied	1027 (16.0)	90 (8.3)	
Dissatisfied	228 (3.6)	29 (2.7)	
Very Dissatisfied	113 (1.8)	2 (0.0)	
**Satisfaction with relationship with father**			< 0.001
Very satisfied very much	1549 (26.0)	494 (47.2)	
Satisfied	2407 (40.4)	354 (33.8)	
Neither satisfied nor unsatisfied	1233 (20.7)	154 (14.7)	
Dissatisfied	473 (7.9)	35 (3.3)	
Very Dissatisfied	289 (4.9)	10 (1.0)	
**I feel loved and cared for by my parents**			< 0.001
Always	2555 (39.3)	706 (64.1)	
Often	1961 (30.1)	253 (23.0)	
Sometimes	1668 (25.6)	113 (10.3)	
Rarely	269 (4.1)	20 (1.8)	
Never	56 (0.9)	9 (0.8)	

^a^ *p*-values were calculated by χ2 tests

### Factors associated with smartphone addiction

Table 3 presents crude and adjusted associations between the independent variables (nationality; gender; and connectedness related variables) and smartphone addiction from the pooled sample, and in both Japanese and Thai students separately. In the multivariable analyses, we found that Thai students were almost 2.7 times more likely to be addicted to smartphones than Japanese students (AOR 2.79; 95% CI: 2.37–3.30). Being female had increased odds of smartphone addiction among both among Japanese (AOR 1.53; 95% CI: 1.32–1.78) and Thai students (AOR 1.34; 95% CI: 1.01–1.78). In both Japanese and Thai students, increases in daily average smartphone usage were associated with increased odds of smartphone addiction in a dose-response fashion; 2 to 4 hours [Japanese (AOR 1.69; 95% CI: 1.34–2.14); Thai (AOR 2.14; 95% CI: 1.61–2.85)] and 5 hours or more [Japanese (AOR 5.60; 95% CI: 3.26–9.64); Thai (AOR 5.30; 95% CI: 2.95–9.52)]. Lastly, Japanese students that were less likely to have smartphone addictions were those with parents who regularly noticed when they were unhappy (AOR 0.77; 95% CI: 0.62–0.96), parents who noticed when they did something good (AOR 0.78; 95% CI: 0.61–0.99), and felt love and care from their parents (AOR 0.61; 95% CI; 0.44–0.84) (Table 3).

**Table 3:** Smart phone addiction and family connectedness among Japanese and Thai high school students.

	Total			Japanese students		Thai students	
	n (%)	COR (95% CI)	AOR (95% CI)	COR (95% CI)	AOR (95% CI)	COR (95% CI)	AOR (95% CI)
**Nationality**							
Thai	382 (35.9)	4.13 (3.57–4.78)***	2.79 (2.37–3.30)***	-	-	-	-
Japan	776 (12.0)	1	1				
**Gender**							
Female	711 (18.3)	1.61 (1.42–1.83)***	1.51 (1.31–1.75)***	1.53 (1.32–1.78)***	1.63 (1.35–1.97)***	1.67 (1.29–2.16)***	1.34 (1.01–1.78)*
Male	447 (12.2)	1	1	1	1	1	
*Family connectedness*							
**Frequency of dining with family in the past week**							
Every day	417 (15.5)	1.05 (0.92–1.19)	a	1.03 (0.88–1.20)	a	0.78 (0.60–1.02)	a
Not every day	697 (14.9)	1		1		1	
**I talk to my parents**							
Always	501 (14.8)	0.93 (0.82–1.05)	a	0.83 (0.71–0.96)*	0.82 (0.67–1.00)	0.75 (0.58–0.96)*	0.80 (0.60–1.08)
Not always	650 (15.8)	1		1	1	1	
**My parents noticed when I have a problem/feel unhappy**							
Regularly	613 (14.6)	0.67 (0.59–0.77)***	0.83 (0.70–0.98)*	0.62 (0.53–0.73)***	0.77 (0.62–0.96)*	0.98 (0.75–1.27)	a
Not regularly or never	442 (20.3)	1	1	1	1	1	
**My parents noticed when I do something good**							
Regularly	766 (14.2)	0.58 (0.50–0.67)***	0.70 (0.58–0.84)***	0.56 (0.47–0.68)***	0.78 (0.61–0.99)*	0.79 (0.59–1.05)	a
Not regularly or never	311 (22.3)	1	1	1	1	1	
**Satisfaction with relationship with mother**							
Very satisfied	411 (15.5)	1.02 (0.90–1.16)	a	0.79 (0.67–0.93)**	0.92 (0.72–1.19)	0.76 (0.59–0.98)*	1.06 (0.74–1.52)
Other	687 (15.2)	1		1	1	1	1
**Satisfaction with relationship with father**							
Very satisfied	311 (15.5)	1.03 (0.89–1.19)	a	0.83 (0.68–0.99)***	0.92 (0.72–1.19)	0.73 (0.56–0.94)*	0.85 (0.61–1.20)
Other	736 (15.1)	1		1		1	1
**I’ve feel loved and cared for by my parents**							
Always	496 (15.5)	1.02 (0.90–1.16)	a	0.88 (0.75–1.03)	a	0.57 (0.44–0.74)***	0.61 (0.44–0.84)**
Not always	652 (15.3)	1		1		1	1
**Smartphone usage per day (average/day)**							
5 hours and more	387 (29.4)	5.45 (4.45–6.67)***	3.54 (2.81–4.46)***	3.31 (2.59–4.23)***	3.49 (2.62–4.66)***	5.60 (3.26–9.64)***	5.30 (2.95–9.52)***
2–4 hours	613 (15.0)	2.36 (2.04–2.73)***	1.74 (1.47–2.06)***	1.57 (1.29–1.91)***	1.69 (1.34–2.14)***	2.09 (1.60–2.74)***	2.14 (1.61–2.85)***
One hour or less	152 (7.1)	1	1	1	1	1	1

* significant *p-*values < 0.05

** significant *p-*values < 0.01

*** significant *p-*values < 0.001; COR: Crude Odds Ratio; AOR: Adjusted Odds Ratio. The following covariates were included in the models based on their statistical significance level (p-value < 0.05) in the bivariate analysis covariates: nationality; gender; frequency of dining with family in the past week; frequency of talking with parents; “How often your parents notice when you have a problem/feel unhappy”; Frequency with which your parents let you know that you did something good”; satisfaction with relationship with mother; satisfaction with relationship with father; perceived love and care from parents; and smartphone usage. a: not included in multivariable analysis

## Discussion

This study is the first to present a comparison of smartphone addiction prevalence and associated factors in two Asian countries of differing economic development and socio-cultural characteristics. Our findings are consistent with results from previous research showing high prevalence of smartphone ownership among high school students in developed and developing settings [9, 31, 35, 47].

We found a higher prevalence of smartphone addiction among Thai students than among Japanese high school students, with Thai students being almost 3 times more likely than Japanese students to be addicted to smartphones. Although this finding might be real and reflect a true difference in the prevalence of smartphone addiction between the two countries, it is important to underscore other factors that might explain the documented difference in the prevalence of smartphone addiction. Firstly, there is a time lag of 2 years between the two surveys. The prevalence of smartphone use which is known to be time-sensitive (generally increasing through time) could possibly explain the higher prevalence of smartphone addiction among Thai students. Secondly, while the survey in Japan was conducted almost country wide, the Thailand survey was limited to Chiang Mai, a Northern province in Thailand. Thirdly, the difference could also be attributed to differing levels of parental restriction on smartphone usage between the two countries. As observed in this study, a higher proportion of Japanese parents employed restrictive measures including filtering programs and limited monthly packages for Internet use than Thai parents.

In this study, female students were at higher risk of smartphone addiction than male students. This is consistent with previous studies on smartphone addiction and other forms of smartphone overuse among adolescents and young people [9, 11, 33, 35–38, 48]. The reason for this gender difference remains unclear; however, previous studies have shown different patterns of smartphone usage between males and females, with females tending to show greater use of smartphones and more addiction to social media (almost all smartphones come equipped with social media apps). However, males spend more time gaming (mostly on laptops or personal computers) and are more addicted to gaming than females [14, 48]. Other studies provide contradictory findings regarding gender differences. For example, Buctot et al. found a higher prevalence of smartphone addiction in adolescent males compared to females in the Philippines [28]. Similarly, males were found to be more prone to smartphone [49] and Internet addiction [50, 51] compared to females.

Overall, Thai students appeared to be more connected to family than Japanese students in this study. A higher prevalence of Thai students reported dining with their family every day; always talking to their parents; being very satisfied with relationships with both parents; and always feeling love and care from both parents. Evidence from Japan suggests that the proportion of children eating alone during mealtimes began to increase in the early nineties, and was estimated at 30% among third year middle school students [52]. This increased rate of children eating alone has reduced opportunities for family conversation, and has possibly weakened family connectedness in Japan. However, parental involvement was more pronounced in Japanese high students than in Thai students. Parental involvement was also associated with smartphone addiction but only among Japanese high school students; students with parents who regularly noticed when they had problems or when they did something good were less likely to have smartphone addiction. Similarly, perception of parental love and care was associated with lower likelihood of smartphone addiction in Japanese students. While parental involvement was protective against smartphone addiction among the Japanese, the overall domain of family connectedness was a better indicator, and the rate of smartphone addiction was higher in Thai adolescents.

This study has several limitations that are worth considering. First, the cross-sectional design precludes drawing causal inferences among study variables. Second, the limited number of variables included in the models mean that we might have missed important variables associated with smartphone addiction in these populations due to the limited socio-demographic data collected. Factors such as depression and anxiety [7, 8, 14, 19, 20, 33], substance use (alcohol use and smoking) [19, 33, 35], self-esteem [34], poor sleep quality [12, 14, 20], and low academic performance [7, 9, 27] have been associated with smartphone addition. Third, the specific context of the two research teams made it difficult to collect data using the same criteria. The survey in Japan employed quota sampling and collected data on a national level, whereas the sample in Thailand was collected from a single province (Chiang Mai) due to limited logistical capacity, resulting in different sample sizes. Also, the sample in Japan was obtained in 2014, while data collection in Thailand took place in 2016. These differences might influence the results of this study. Fourth, there is the possibility of recall bias for the measurement of smartphone usage. Lastly, we did not use a validated scale to capture family connectedness. It is unclear to what extent this might affect the results of the current study. Despite the above limitations, this study remains the first of its kind to provide a comparative analysis of smartphone addiction between high school students in Japan and Thailand.

## Conclusion

Although there was high smartphone ownership in both Japanese and Thai adolescents, smartphone addiction was more prevalent among Thai adolescents. Females in both countries were more likely than males to have a smartphone addiction. Parental involvement appeared to have a protective effect against smartphone addiction, particularly among Japanese adolescents. Our results suggest that interventions for reducing smartphone addiction should take into account both context and gender, and should leverage the protective effect of parental involvement. Future students can build upon this study by clarifying and validating the results.

## Supporting information

S1 Data (SAV)
